# Constipation of the Bowels during Twenty-One Days, Successfully Treated

**Published:** 1827-12

**Authors:** John Jones

**Affiliations:** Member of the Royal College of Surgeons.


### CONSTIPATION.

CASE I. *Constipation of the Bowels during twenty-one days, successfully treated.* By JOHN JONES, Esq. Member of the Royal College of Surgeons.

THE following case of protracted constipation appears Interesting from the long continuance of the obstruction, the painful symptoms with which it was accompanied, and its successful termination after resisting the most powerful means for twenty-one days. If you consider it worthy a place in your valuable Journal, I shall feel obliged by its insertion.

Mary Swindell, ætat. between fifty and sixty, mother of eighteen children, rather corpulent, of a sallow and unhealthy complexion; her general health tolerably good, excepting that she is occasionally affected with colic from constipation. Of late her bowels have been regular; but, on Friday the 24th of August, she was attacked with spasmodic pains, accompanied with sickness. I saw her on the Monday following, and found that her bowels had not been moved since the preceding Friday morning, although she had taken in succession a dose of castor-oil, an ounce of Epsom salts, and a scruple of Extract. Colocynth. Comp. She complained of occasional severe pains of the abdomen, which were not, however, increased upon pressure. Tongue furred; pulse natural. I bled her ad ℥xij.; ordered fomentations to the abdomen, a drop of croton-oil to be given immediately, and repeated in two hours if the bowels were not relieved.

28th.—Pain less frequent, and sickness abated; in other respects the same as yesterday. The following pills were prescribed—

℞. Submur. Hydrarg. gr. xij.; Pulv. Jalap. ℈ij.; Syrup. q. s. ut fiat massa, dividend in pil. xij. Capiat ij. quaque quarta horâ.—An enema, with soft soap dissolved in warm water, to be injected in as large a quantity as can be retained, and repeated two or three times a-day.

29th.—Medicines produced no effect, excepting that a few scybalæ followed one of the injections.

Repet^r Enema, cui adde Ol. Ricini ℥ij.

30th.—Symptoms the same.

A turpentine injection was given, and the enemata with soft soap continued.

31st.—Still unrelieved.

Four drops of croton-oil to be given in divided doses.

Sept. 1.—Croton-oil produces uneasiness and restlessness, but no effect upon the bowels. The following mixture was ordered—

R. Sulph. Magnes. ℥ij.; Inf. Sennæ ℥ vj.; Tinct. Jalap. ℥ss. M. capiat cochl. iij. quaque secunda horâ.

2d.—Complains of great uneasiness and fulness of epigastrium.

An enema with infusion of tobacco administered, which produced considerable depression, but no evacuation, excepting a few hard scybalæ.—Six drops of Croton-oil made into three pills, one to be taken every two hours.

3d.—Pills were returned by vomiting. A tobacco-smoke enema was administered, after which she was more composed: it was repeated in the course of the day.

4th.—Free from pain, excepting uneasiness at the scrobiculus cordis.

An emulsion with two ounces of Castor-oil to be given in divided doses.—Tobacco-smoke injection repeated.

5th and 6th.—She continued free from pain, but was occasionally sick.

Enemata with soft soap frequently administered, but without producing any evacuation.

7th.—Uneasiness and fulness of epigastrium increased, accompanied with much sickness and vomiting of an acid liquor; tongue greatly loaded; pulse about ninety, and rather hard. Fearing the supervention of inflammation, I again bled her ad ℥xij., and ordered the tobacco-smoke injection to be repeated.

In the evening of this day, another surgeon accompanied me to see her. The whole of the abdomen was much swollen since the morning: the swelling was elastic, resisting, and, when struck, sounded hollow, evidently indicating the existence of tympanitis. We considered the case hopeless. The following pill was given at bedtime—

R. Submur. Hydrarg. gr. vj.; Pulv. Opii gr. j. M.

8th.—Slept tolerably well; is free from pain; sickness less urgent.

Repet^r Pilula, cui adde Extr. Colocynth C. gr. viij.

10th.—Sickness has returned with greater violence; the stomach will retain nothing. What is vomited has the appearance of coffee-grounds.

Dr. Forester, senior physician to the General Infirmary of this place, was requested to see her. He considered the swelling of the abdomen tympanitic, and that, unless the bowels were relieved, she could not long survive. He prescribed an injection with three drops of Croton-oil, pills with Socotrine Aloes alone, and the Effervescing Draught, with Tincture of Hops and Syrup of Poppies, to relieve the irritability of the stomach.

11th.—Injection produced no effect. Sickness was relieved by Dr. Forester's prescription, but has again returned, accompanied with vomitings of the same coffee-ground appearance. She sleeps much, and refuses to take more medicine.

12th.—Symptoms unabated. Complains of great uneasiness at the scrobiculus cordis; tongue greatly furred; swelling of the abdomen increased, and more tense; coffee-ground vomitings continue; pulse quick and feeble. She entirely despairs of recovery; her friends also have given her up, and are anxiously expecting her dissolution. As a last effort, I prescribed the following liniment, to be well rubbed upon the abdomen, particularly over the region of the stomach and arch of the colon, every two or three hours, and each rubbing to be continued for at least twenty minutes.

℞. Camphoræ ʒj.; Ol. Oliv. Opt. ℥jss.; Liq. Ammon. ℥ss. M.

13th.—Liniment has been used, but not so frequently nor assiduously as directed. Ordered to be continued with more regularity.

In the evening of this day, the twenty-first from the commencement of the attack, the nurse brought me the unexpected and gratifying information that our patient was at length relieved. About half an hour after friction with the liniment had been well used, she had a most copious liquid evacuation, which was soon followed by others equally copious, having the consistence and appearance of yeast. I immediately visited her, and found a great alteration for the better. The poor woman seemed overpowered with feelings of gratitude; all her painful symptoms had vanished; the swelling of the abdomen had entirely subsided; the sickness and oppression at the stomach were removed; pulse resumed its natural quickness and strength; and she expressed her general feelings by emphatically exclaiming "I am now in heaven!"

From this time no unpleasant symptoms recurred, and she now enjoys her usual health.

*Derby; October 8th*, 1827.

